# Clinical and Gene Features of SARS-CoV-2-Positive Recurrence in Patients Recovered From COVID-19

**DOI:** 10.3389/fmolb.2022.875418

**Published:** 2022-06-08

**Authors:** Yuying Peng, Shaoqi Wang, Ruihuan Chai, Yong Chen, Nan Li, Boning Zeng, Qian Tang, Kai Zheng, Youfang Liang, Shouxia Xie, Wei Huang, Shaoxiang Wang, Xiao Wang

**Affiliations:** ^1^ Department of Pharmacy, Shenzhen Key Laboratory of Prevention and Treatment of Severe Infection, Shenzhen People’s Hospital (The Second Clinical Medical College, Jinan University; The First Affiliated Hospital, Southern University of Science and Technology), Shenzhen, China; ^2^ School of Pharmacy, Jinan University, Guangzhou, China; ^3^ Department of Internal Medicine, Hubei Province Corps Hospital of The Chinese Armed Police Force (CAPF), Wuhan, China; ^4^ School of Pharmaceutical Sciences, Shenzhen University Health Science Center, Shenzhen, China; ^5^ Chinese PLA Center for Disease Control and Prevention, Beijing, China; ^6^ Bacteriology and Antibacterial Resistance Surveillance Laboratory, Shenzhen Institute of Respiratory Diseases, Shenzhen People’s Hospital (The Second Clinical Medical College, Jinan University, The First Affiliated Hospital, Southern University of Science and Technology), Shenzhen, China

**Keywords:** COVID-19, SARS-CoV-2, re-positive, immune, inflammation

## Abstract

There are still frequent reports that a number of recovered coronavirus disease 2019 (COVID-19) patients following discharge have re-detectable positive (RP) results by RT-PCR. Understanding the clinical and molecular characteristics of RP patients may have implications for curbing the COVID-19 pandemic. In this study, 318 COVID-19 convalescent patients, including 59 RP patients and 259 non-RP (NRP) patients, were enrolled. Among RP patients, women accounted for a significantly high proportion (67.8%), and the titers of IgG and IgM antibodies in this group were also significantly high. Differentially expressed genes (DEGs), including 692 upregulated and 383 downregulated genes, overlapped in two public GEO datasets containing RP and NRP blood cell samples. Enrichment analysis indicated that these DEGs were related to several key signaling pathways, such as viral infection, immune activation, and inflammatory responses. Importantly, 59 indicator genes constituting the core network exhibited high diagnostic values and were correlated with markers of different immune cells. Among these, 12 drug-related genes were associated with the RP results. Our work suggests that, in addition to clinically available features, blood cell transcriptome sequencing can be performed to obtain gene signatures for diagnosis of RP patients.

## 1 Introduction

The emerging acute respiratory disease, widely known as coronavirus disease 2019 (COVID-19), is a highly infectious pandemic caused by coronavirus SARS-CoV-2 infection, which has spread across the world (Wang et al., 2020). Patients infected with SARS-CoV-2 exhibit clinical symptoms including fever, dry cough, and fatigue (Guan et al., 2020; Miró et al., 2021). As of this writing, the pandemic has caused more than 200 million people to be infected, as well as four million deaths (https://covid19.who.int).

Currently, most COVID-19 studies concentrate on clinicopathological and epidemiological characteristics, as well as laboratory tests and efficacy results in infected patients (Chan et al., 2020; Lan et al., 2020; Pan et al., 2020; Wang et al., 2020), leading to a deeper understanding of patients with initial viral infection (Bradley et al., 2020; Liu et al., 2020; Song et al., 2020). Many other researchers have investigated the important roles of innate and adaptive immune responses in SARS-CoV-2 infection and the pathogenesis of COVID-19 (Paces et al., 2020; Zhang et al., 2021). It is believed that production of a large amount of inflammatory cytokines, such as interleukin (IL)-6, interferon (IFN)-γ, and tumor necrosis factor (TNF)-α, known as the “cytokine storm,” correlates with COVID-19 infection (Ragab et al., 2020). In addition, B cells recognize viral proteins and are activated to produce SARS-CoV-2-specific antibodies (Zhang et al., 2020). In addition, the humoral response to SARS-CoV-2 initiates the release of specific immunoglobulins G and M (IgG and IgM) (Wehbe et al., 2021).

One emerging problem is that there are several COVID-19 patients with re-detectable positive (RP) results after treatment (Zhou et al., 2020; Huang et al., 2021). Because of their potential infectivity, more attention should be given to RP patients (Tay et al., 2020). Judging from the strict hospital management of RP patients at that time, it was likely that they have a latent infection not detectable temporary from the tests. Previously, Gao et al. (2020). illustrated that the Nucleic acid results might be influenced by patients’ condition, which lead to temporarily undetectable by RT-PCR tests. For patients’ condition, it might be related to underlying diseases and treatment methods. Patients with underlying diseases, such as diabetes and hypertension, once infected with SARS-CoV-2, would have a further weakened in immune system, ultimately, who are more likely to relapse after discharge (Hussain et al., 2020). In addition, the COVID-19 patients were treated with antiviral drugs that clear SARS-CoV-2, whose immune function will be influenced. However, when the antiviral therapy was discontinued, the virus would be activated due to a decreased immune response (Balachandar et al., 2020; Wu et al., 2020). Due to these potential reasons, patients with RP results require longer isolation periods and more frequent nucleic acid tests, leading to a greater burden on hospitals and patients. Therefore, it is critical to explore the internal mechanisms and molecular features of patients with RP to identify sensitive and reliable indicators for RP patient diagnosis.

In this study, we focused on the risk associated with clinical characteristics, related signaling pathways, and indicator genes to understand the clinical and molecular features of patients with RP. We collected and analyzed the epidemiological and laboratory characteristics of 318 COVID-19 convalescent patients, including 59 RP patients. A systematic literature survey on the clinical features was conducted to comprehensively evaluate the application value of these clinical characteristics. We also analyzed two GEO datasets containing RP and NRP samples; differentially expressed genes (DEGs) were enriched in signaling pathways associated with viral infection, immunity, and inflammation in patients with RP. In addition, we performed correlation analysis and identified many high-standard diagnostic indicator genes in RP patients, which can be used as indicative biomarkers for the recurrence of positive SARS-CoV-2.

## Materials and Methods

### Clinical Information and Data Collection

This study, conducted at the Wuhan Rehabilitation Station of Hubei University, enrolled 318 patients who were instructed to undergo isolation observation from 31 January to 26 March, 2020. Epidemiological information and laboratory test results were also obtained. A total of 318 patients were divided into two groups: 59 patients with RP and 259 patients with NRP. Written informed consent was obtained from all patients, and all methods were performed in accordance with relevant safety guidelines.

### Statistical Analysis

Categorical variables were presented as absolute and relative frequencies (%), and continuous variables were presented as median and interquartile range (IQR) and number (%). A univariate logistic regression model was used to compute odds ratio (OR) and *p*-values. Spearman’s correlation analysis was used to analyze the correlation between categorical and continuous variables. All statistical results were analyzed using IBM SPSS statistics version 23.0, and the results were plotted using GraphPad Prism version 7.0.

### Acquisition and Analysis of Re-Detectable Positive Patient Datasets

Two microarray datasets of patients with RP and NRP were obtained from the GEO database (https://www.ncbi.nlm.nih.gov/geo/). The GSE166253 expression profile contained six NRP samples and 10 RP samples derived from the University of Science and Technology of China. The GSE179627 expression profile included 11 NRP and 15 RP samples from the College of Bioinformatics in China. These samples were used for analysis of gene transcription of their peripheral blood mononuclear cells (PBMCs). Based on the expression levels of all genes in the samples, the correlation between samples were assessed by SangerBox (http://sangerbox.com/), and followed by Pearson correlation analysis. |Cor| ≥ 0.9 was clustered and |Cor| < 0.9 was excluded. Sample GSM5425261 is abnormal and has relatively low correlation with others (|Cor| < 0.9) (Supplementary Figure S1). So, it was removed from the database.

### DEG Analysis

The adjusted *p*-value < 0.05 and |logFC| > 0.5 were used to define DEGs identified by the limma package in R language (version 4.0.3), which classified expression differences between patients with RP and NRP by analyzing raw data from public GEO databases. DEGs were then visualized by volcanic map. The overlapping genes between GSE166253 and GSE179627 were analyzed using Venn diagram on the website (http://bioinformatics.psb.ugent.be/webtools/Venn/).

### Pathway Enrichment Analysis

The functions of the DEGs were ascertained by Gene Ontology (GO) annotation and Kyoto Encyclopedia of Genes and Genomes (KEGG) pathway analysis using the Estimate package in R (version 4.0.3). Significant pathway terms (*p* < 0.05) in GO and KEGG were enriched, and the top 10 enrichment terms related to viral infection, immunity, and inflammation were presented with bubble diagrams using Sangerbox (http://www.sangerbox.com/Tool).

### Co-expression Network Construction

Spearman correlation analysis was used to analyze the correlation between the RP results and the level of DEGs. In two RP-related GEO datasets, we performed WGCNA analysis with high correlation and low node count (power = 1, weight = 0.75) to obtain a co-expression network with highly accurate diagnostic indicator genes. Overlapped indicator genes were selected, and their networks were drawn using Cytoscape (http://www.cytoscape.org/; v3.7.1).

### Correlation Analysis Between Indicator Genes and Immune Markers

The relations between indicator genes and immune markers were examined using Pearson correlation analysis via the R package. In Scatter diagram, |Cor| ≥ 0.4 and *p* < 0.05, which were consistent in both two datasets, were considered statistically significant.

### Mining Clinically Relevant Drug-Gene Interactions

Drug-gene interactions were analyzed using the drug-gene interaction database (DGIdb, https://dgidb.org/).

## Results

### Risk Factors Associated With Re-Detectable Positive Results

**FIGURE 1:**
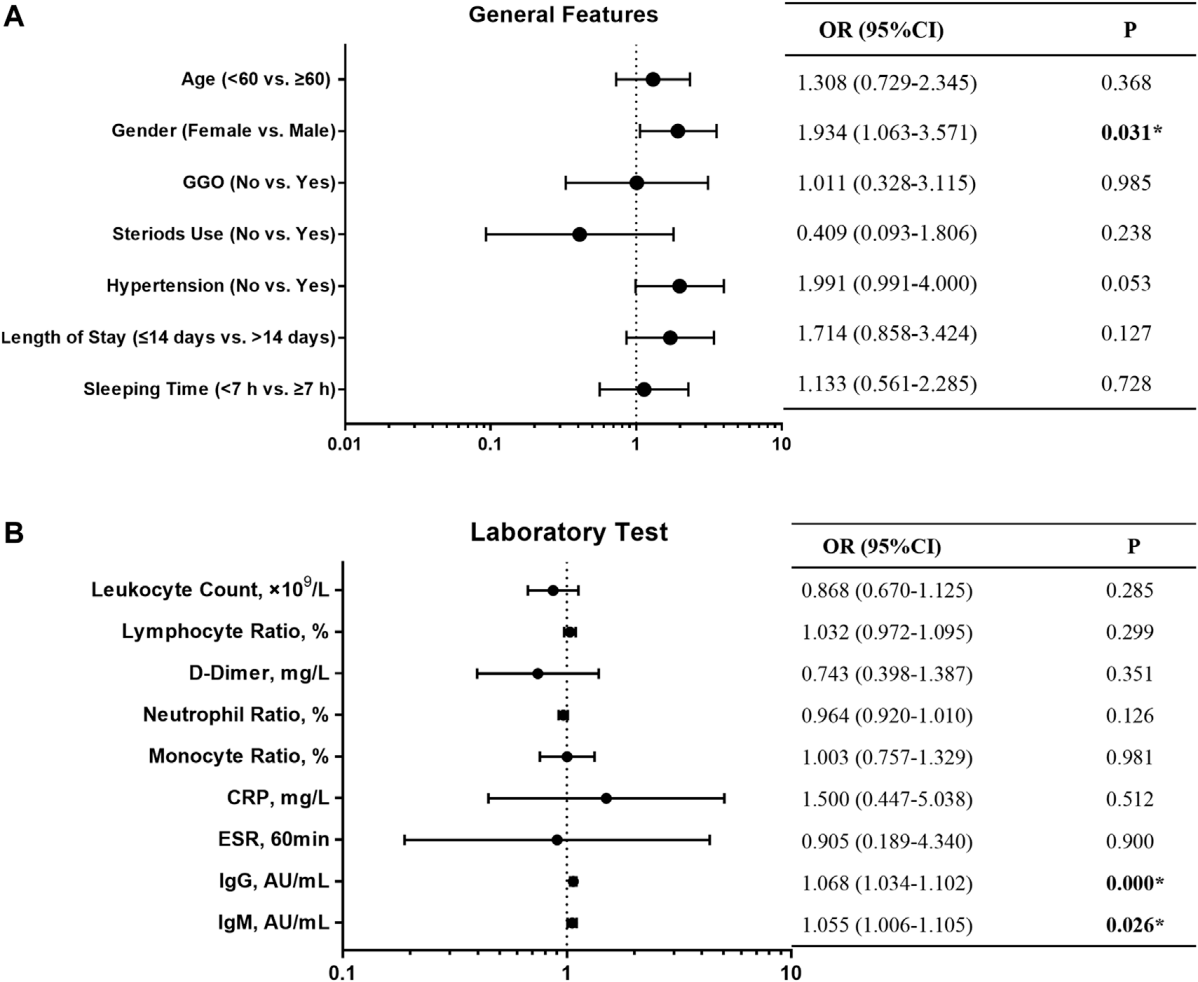
Forest plot of the OR, 95% CI of OR, and P value of the general features **(A)** and laboratory test **(B)** included in the model.

We followed 318 COVID-19 convalescent patients and analyzed their demographic and clinical characteristics. Fifty-nine patients tested positive again (RP rate, 18.6%), and their general features are shown in Table 1. A lower number of men (32.2%) turned RP than women (67.8%). Univariate logistic regression analysis was employed to identify the risk factors associated with RP results. OR with a confidence interval (CI) of general features and laboratory tests was obtained, and the results are shown as forest plots (Figure 1). Consequently, the logistic regression indicated that sex and IgG and IgM titers influenced the risk of RP. Women had a higher risk of having an RP result than men (OR 1.934, 95% CI: 1.063–3.571, *p* = 0.031). The inflammatory markers CRP (OR 1.500, 95% CI: 0.447–5.038, *p* = 0.512) and ESR (OR 0.905, 95% CI: 0.189–4.340, *p* = 0.900) were not statistically different between NRP and RP groups. Recovered COVID-19 patients who had a higher titer of IgG and IgM in their isolation period after discharge had a higher risk of RP (OR 1.068, 95% CI: 1.034–1.102, *p* = 0.000; OR 1.055, 95% CI: 1.006–1.105, *p* = 0.026, respectively).

**TABLE 1:** Epidemiological characteristics of COVID-19 patients with and without RP results.

Variables	Total (*N* = 318)	NRP (*N* = 259)	RP (*N* = 59)
Total (n)	318	259 (81.4%)	59 (18.6%)
Age (Years)	54 (43–63)	57 (46–64)	54 (43–62)
<60	210 (66.0%)	174 (67.2%)	36 (61.0%)
≥60	108 (34.0%)	85 (32.8%)	23 (39.0%)
Gender			
Female	175 (55.0%)	135 (52.1%)	40 (67.8%)
Male	143 (45.0%)	124 (47.9%)	19 (32.2%)
GGO Nodule			
No	22 (6.9%)	18 (6.9%)	4 (6.8%)
Yes	278 (87.4%)	227 (87.6%)	51 (86.4%)
Missing	18 (5.7%)	14 (5.4%)	4 (6.8%)
Steroids Use			
No	270 (84.9%)	217 (83.8%)	53 (89.8%)
Yes	22 (6.9%)	20 (7.7%)	2 (3.4%)
Missing	26 (8.2%)	22 (8.5%)	4 (6.8%)
Hypertension			
No	269 (84.6%)	224 (86.5%)	45 (76.3%)
Yes	49 (15.4%)	35 (13.5%)	14 (23.7%)
Length of Stay			
≤14 days	91 (28.6%)	79 (30.5%)	12 (20.3%)
>14 days	213 (67.0%)	169 (65.2%)	44 (74.6%)
Sleeping Time			
<7 h	121 (38.1%)	103 (39.8%)	18 (30.5%)
≥7 h	115 (36.2%)	96 (37.1%)	19 (32.2%)
Missing	82 (25.8%)	60 (23.2%)	22(37.3%)
			
Laboratory Test			
Leukocyte Count, ×10^9^/L	5.3 (3.7-6.8)	5.4 (3.8-6.9)	5.0 (3.6-5.8)
<4	23 (7.2%)	17 (6.6%)	6 (10.2%)
[4-10]	45 (14.2%)	39 (15.1%)	6 (10.2%)
>10	9 (2.8%)	8 (3.1%)	1 (1.7%)
Lymphocyte Ratio, %	22.7 (14.5–30.7)	20.1 (14.1–30.1)	28.6 (14.9–34.0)
<20	22 (6.9%)	19 (7.3%)	3 (5.1%)
[20-40]	22 (6.9%)	16 (6.2%)	6 (10.2%)
>40	5 (1.6%)	3 (1.2%)	2 (3.4%)
D-Dimer, mg/L	0.7 (0.2–2.5)	0.6 (0.2–2.9)	0.8 (0.2–1.5)
<0.5	7 (2.2%)	5 (1.9%)	2 (3.4%)
≥0.5	25 (7.9%)	20 (9.7%)	5 (8.5%)
Neutrophil Ratio, %	66.6 (56.1–77.5)	70.4 (57.6–78.2)	57.8 (48.0–74.8)
<40	1 (0.3%)	0 (0%)	1 (1.7%)
[40-70]	41 (12.9%)	32 (12.4%)	9 (15.3%)
>70	24 (7.5%)	21 (8.1%)	3 (5.1%)
Monocyte Ratio, %	10.1 (6.6–10.9)	10.2 (6.2–10.8)	9.8 (7.9–11.0)
≤8	12 (3.8%)	8 (3.1%)	4 (6.8%)
>8	21 (6.6%)	14 (5.4%)	7 (11.9%)
CRP, mg/L	5.67 (3.93–16.18)	5.67 (3.95–31.4)	5.755 (3.695–11.65)
≤10	39 (12.3%)	26 (10.0%)	13 (22.0%)
>10	20 (6.3%)	15 (5.8%)	5 (8.5%)
ESR, 60min	28 (16–42.15)	28.5 (14.5–42.0)	22 (17.5–50.5)
<20	14 (4.4%)	10 (3.9%)	4 (6.8%)
≥20	24 (7.5%)	18 (6.9%)	6 (10.2%)
Antibody, AU/mL			
IgG	89.0 (58.5–157.6)	60.5 (43.4–77.2)	154.9 (117.8–181.8)
	72 (22.6%)	35 (13.5%)	37 (62.7%)
IgM	5.2 (2.3-17.8)	3.9 (1.4-6.9)	8.7 (3.6-28.8)
	72 (22.6%)	35 (13.5%)	37 (62.7%)

CRP, C-reactive protein; ESR, erythrocyte sedimentation rate.

There were no substantial differences in other general features or laboratory findings between the RP and NRP patients. In addition, for comprehensive comparison and analysis, clinical characteristics of patients with RP reported in previous studies are listed in Table 2, including age, LYM count, IL-6, AST, and D-dimer. Unexpectedly, most of the risk factors listed in the table are inconsistent with our results.

**TABLE 2:** The clinical features associated with RP results reported in recent literature.

Samples (NRP:RP)	Rate (%)	Clinical characteristics (*p* < 0.05)	References
17,814:2466	12.16	Age, genderetc.	Wu et al. (2021)
682:60	8.09	Age, Lymphocyte countetc.	Dong et al. (2021)
588:157	21.07	Age, LYM etc.	Luo et al. (2021)
532:87	14.05	Age, clinical classificationetc.	Lu et al. (2020)
345:69	16.67	Interleukin 6, lactate dehydrogenase, AST, LMY, immunoglobulin protein, BMI etc.	Huang et al. (2020)
345:23	6.25	Lymphocyte, AST, LDH, D-Dimer, CRPetc.	Zhou et al. (2020)
232:92	28.40	Gender, positive IgM, negative IgGetc.	Liu et al. (2021)
132:13	8.97	IgG, longer virus shedding durationetc.	Hong et al. (2021)

### Identification of Differentially Expressed Genes Between Re-Detectable Positive and Non-RP Patients

**FIGURE 2:**
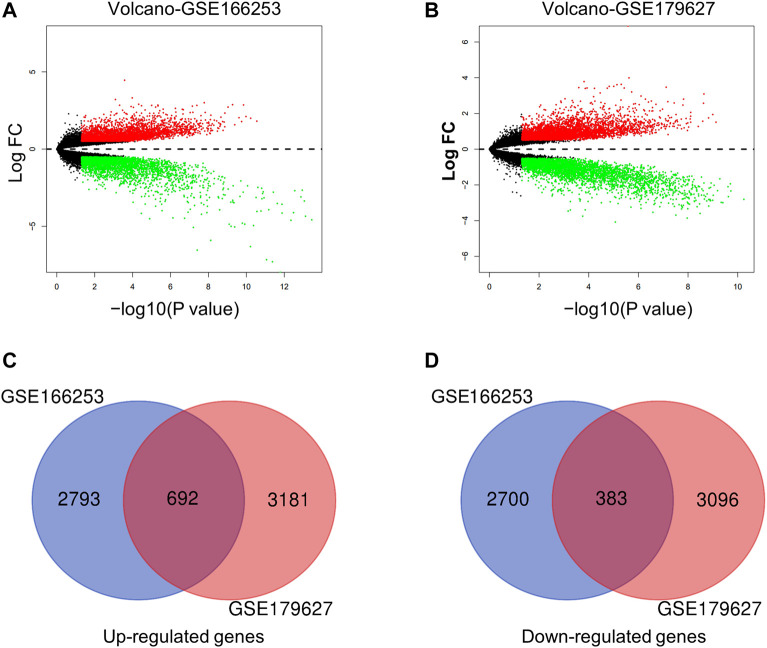
Characteristics of differentially expressed genes (DEGs) between RP and NRP samples. **(A,B)** Volcano plots of mRNAs in two datasets (GSE166253 and GSE179627) respectively. The red and green dots indicate the up-regulated and down-regulated differentially mRNAs, respectively. 692 upregulated genes **(C)** and 383 downregulated genes **(D)** were showed in the overlapping Venn diagram. The blue circle indicates GSE166253, and the red circle indicates GSE179627.

Next, we conducted a more specific analysis at the gene level to identify more accurate signatures for patients with RP. DEGs from the two datasets were screened using filter criteria. In the GSE166253 dataset, 3,485 upregulated and 3,083 downregulated mRNAs were identified in RP patients compared to NRP patients (Figure 2A). Similarly, in the GSE179627 dataset, 3,873 upregulated mRNAs and 3,479 downregulated mRNAs were identified (Figure 2B). Moreover, we found 1,075 overlapping DEGs within the two datasets, including 692 upregulated and 383 downregulated genes, as shown in the Venn diagram (Figures 2C,D).

### Functional Annotation of Differentially Expressed Genes

**FIGURE 3:**
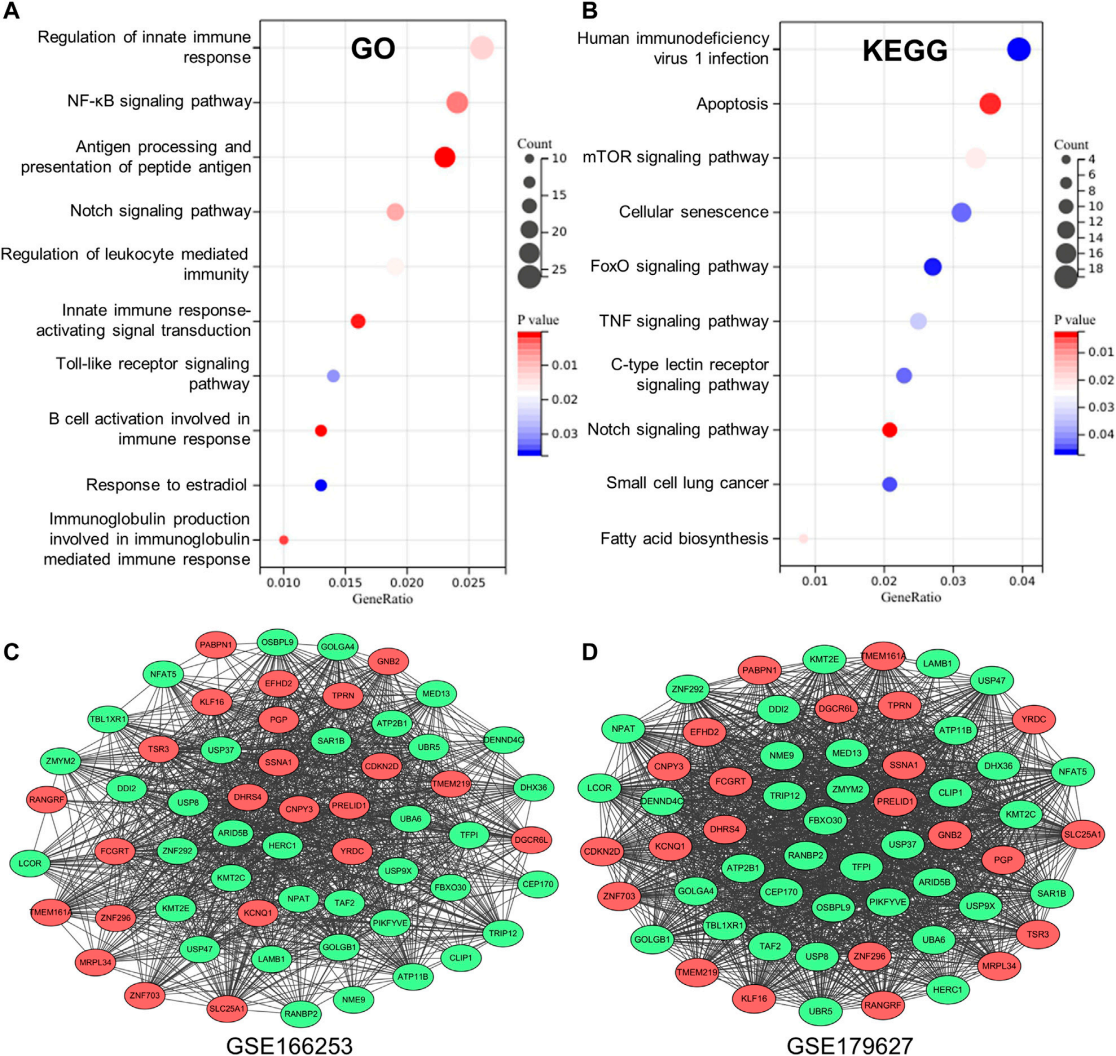
Establishment of database core network. **(A)** GO enrichment analysis of the overlapping DEGs in biological process (BP) subgroup and the genes enriched on the pathway. **(B)** KEGG enrichment analysis of the overlapping DEGs the genes enriched on the pathway. **(C,D)** 59 genes with high correlation coefficient in the two RP-related GEO datasets is visualized in Cytoscape.

To obtain key information about the biological function of the 1,075 overlapping DEGs between RP and NRP patients, we performed GO and KEGG enrichment analyses. The results of GO annotation analysis indicated that DEGs were significantly enriched in terms of regulation of innate immune response, regulation of NF-κB signaling, antigen processing, and presentation of peptide antigen in the BP subgroup (Figure 3A). In addition, the most significant KEGG pathways were enriched for overlapping DEGs in COVID-19 patients, including human immunodeficiency virus 1 infection, apoptosis, the mTOR signaling pathway and TNF signaling pathway (Figure 3B). In addition, to detect the level of inflammation in RP patients, we analyzed the inflammatory markers TNF and IFNG which were considered to mediate the development of inflammation (Han et al., 2020; Montalvo Villalba et al., 2020). We found that compared with the NRP groups, the expression levels of TNF and IFNG in the RP groups were higher (Supplementary Figure S2). These DEGs are closely related to the antiviral immune response and inflammation-related pathways, suggesting that these DEGs may have useful clinical applications in the diagnosis and prevention of COVID-19.

### Co-expression Network Related to Re-Detectable Positive Results

**FIGURE 4:**
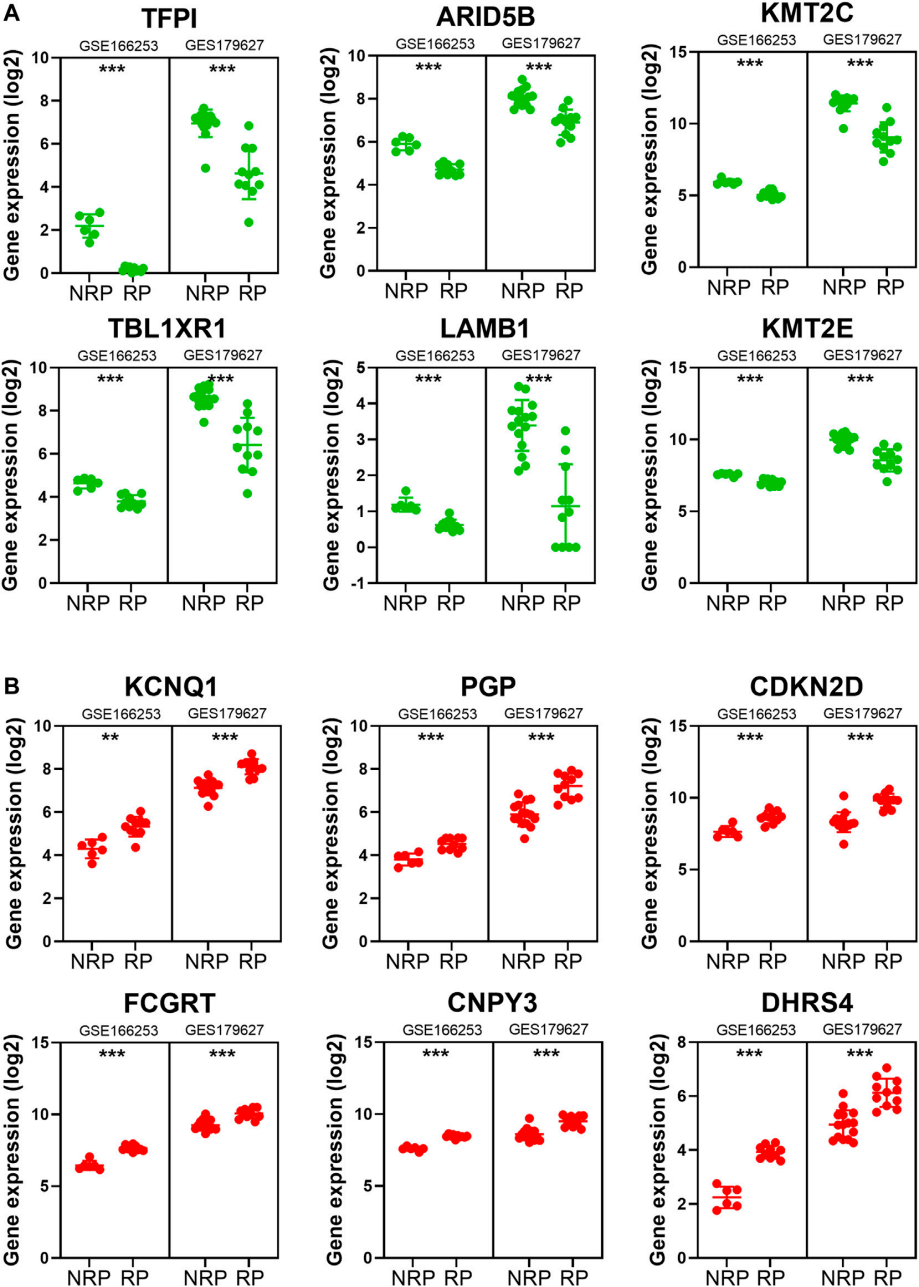
The expression of some indicator genes in the two datasets. **(A)** The down-regulated expression of *ARID5B*, *KMT2C*, *KMT2E*, *LAMB1*, *TBL1XR1* and *TFPI*. **(B)** The up-regulated expression of *CDKN2D*, *PGP*, *KCNQ1*, *FCGRT*, *CNPY3* and *DHRS4*.

To obtain a co-expression network with more and highly accurate diagnostic indicator genes, we performed WGCNA analysis with parameter settings of high correlation and low node count. In the two RP-related GEO datasets, 59 nodes with high correlation coefficients overlapped, and their networks were drawn using Cytoscape (Figures 3C,D). In the network diagram, 23 upregulated genes are represented by red circles, whereas 36 downregulated genes are represented in green. The expression of several key genes is shown in Figure 4, and their correlation with RP results, calculated by univariate logistic regression analysis, is shown in Table 3, which indicates that most of these genes significantly influenced the risk of RP results (*p* < 0.05). Therefore, these DEGs could be used as indicator genes in patients with RP.

**TABLE 3:** Correlation analysis between COVID-19 patients with or without RP results and indicator genes.

Genes	GSE166253		GSE179627		
	Log FC	Cor	Log FC	Cor	P
ARID5B	1.19	−0.84	1.15	−0.784	*
ATP11B	1.06	−0.84	1.68	−0.856	0.987
ATP2B1	1.85	−0.84	2.04	−0.794	*
CDKN2D	1.02	0.784	1.51	0.763	**
CEP170	0.53	−0.784	1.56	−0.856	0.995
CLIP1	0.52	−0.812	1.55	−0.773	0.055
CNPY3	0.86	0.84	0.90	0.773	**
DDI2	0.90	−0.84	1.43	−0.815	0.065
DENND4C	0.66	−0.812	2.17	−0.846	0.157
DGCR6L	1.14	0.84	1.21	0.784	**
DHRS4	1.68	0.84	1.18	0.784	*
DHX36	0.84	−0.784	1.28	−0.856	0.975
EFHD2	0.53	0.812	0.94	0.825	0.050
FBXO30	1.05	−0.84	1.75	−0.784	*
FCGRT	1.21	0.84	0.81	0.763	**
GNB2	0.64	0.84	0.92	0.773	*
GOLGA4	0.67	−0.84	2.37	−0.856	0.989
GOLGB1	1.17	−0.84	1.88	−0.815	0.124
HERC1	0.54	−0.812	1.66	−0.784	**
KCNQ1	1.03	0.756	0.98	0.836	*
KLF16	0.88	0.84	1.24	0.815	**
KMT2C	0.90	−0.84	2.37	−0.815	**
KMT2E	0.55	−0.84	1.43	−0.804	*
LAMB1	0.57	−0.84	2.24	−0.764	*
LCOR	0.71	−0.812	2.08	−0.804	*
MED13	0.53	−0.812	1.76	−0.836	*
MRPL34	0.53	0.84	1.48	0.701	**
NFAT5	0.64	−0.812	1.84	−0.846	0.077
NME9	0.54	−0.756	2.40	−0.743	**
NPAT	0.81	−0.728	2.39	−0.815	*
OSBPL9	0.74	−0.84	1.30	-0.856	0.981
PABPN1	0.52	0.84	0.70	0.753	*
PGP	0.72	0.812	1.32	0.773	*
PIKFYVE	0.76	−0.812	2.03	−0.856	0.993
PRELID1	1.15	0.84	0.97	0.804	*
RANBP2	1.59	−0.84	2.48	−0.794	*
RANGRF	0.93	0.784	1.37	0.794	*
SAR1B	0.97	−0.84	1.49	−0.732	**
SLC25A1	0.85	0.84	1.04	0.836	0.096
SSNA1	0.72	0.84	1.40	0.773	**
TAF2	0.51	−0.84	1.09	−0.825	0.075
TBL1XR1	0.83	−0.84	2.19	−0.804	*
TFPI	2.01	−0.84	2.32	−0.804	**
TMEM161A	1.04	0.84	1.39	0.784	*
TMEM219	0.75	0.84	1.06	0.815	*
TPRN	0.61	0.812	1.21	0.794	*
TRIP12	0.61	−0.84	1.59	−0.815	*
TSR3	0.85	0.84	1.65	0.815	**
UBA6	1.05	−0.84	2.14	−0.846	0.107
UBR5	0.89	−0.84	1.46	−0.784	*
USP37	1.35	−0.84	1.54	−0.804	*
USP47	1.03	−0.84	1.63	−0.763	*
USP8	0.81	−0.84	1.44	−0.773	0.051
USP9X	0.88	−0.84	1.89	−0.784	*
YRDC	0.68	0.84	1.51	0.773	*
ZMYM2	0.52	−0.84	1.54	−0.825	0.101
ZNF292	1.11	−0.812	2.57	−0.773	*
ZNF296	1.19	0.84	1.90	0.742	**
ZNF703	1.59	0.784	1.98	0.804	0.054

Notes: Cor, R-value of Person’s correlation; *p < 0.05, **p < 0.01, ***p < 0.001.

### Correlation Analysis Between Indicator Genes and Immune Markers

**FIGURE 5:**
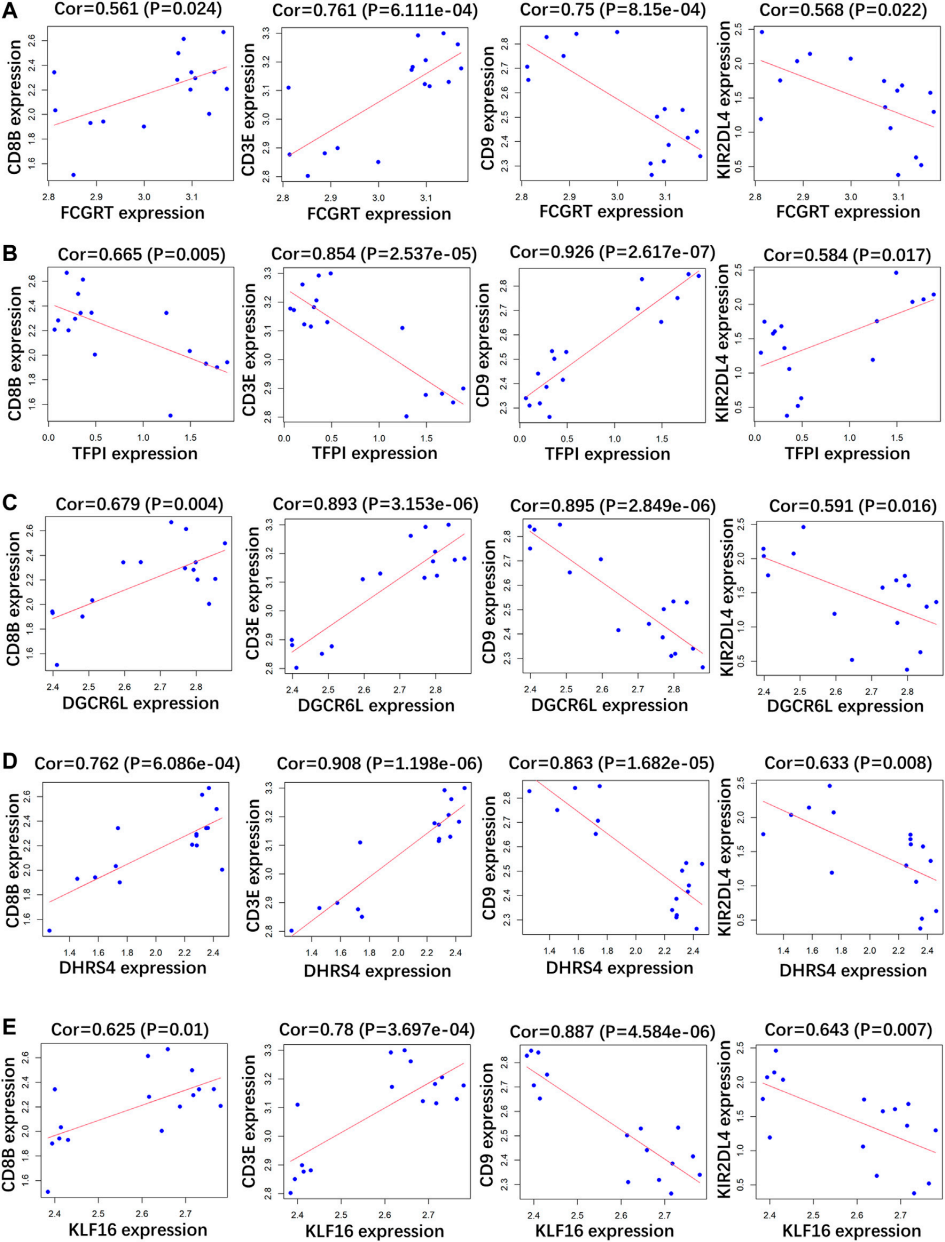
Correlation analysis between **(A)** *FCGRT*, **(B)** *TFPI*, **(C)** *DGCRL6*, **(D)** *DHRS4*, **(E)** *KLF16* and different immune cell markers in GSE166253.

**FIGURE 6:**
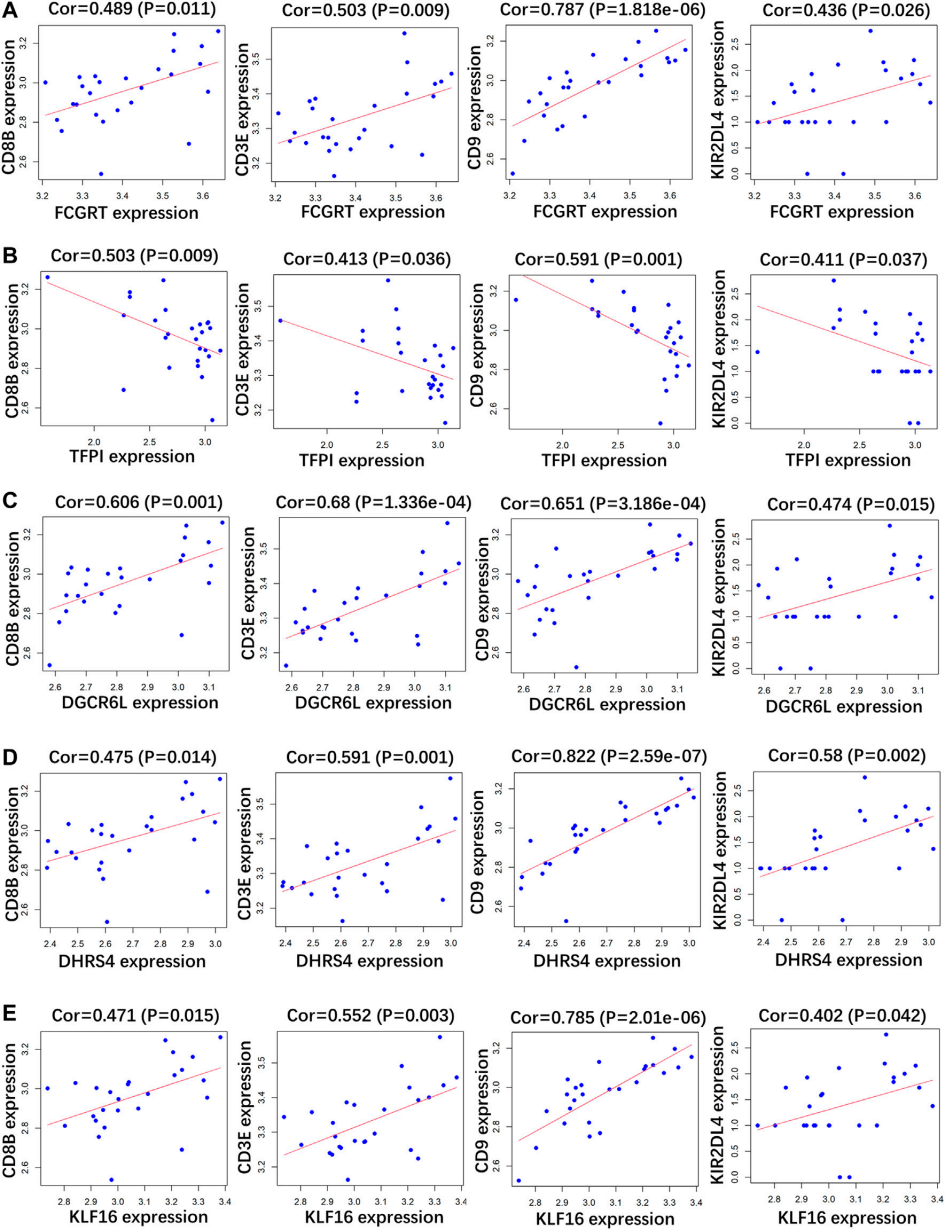
Correlation analysis between **(A)** *FCGRT*, **(B)** *TFPI*, **(C)** *DGCRL6*, **(D)** *DHRS4*, **(E)** *KLF16* and different immune cell markers in GSE179627.

RP patients are usually associated with low autoimmunity; thus, we investigated the correlation between indicator genes and markers of different immune cells. These indicator genes were significantly correlated with hallmarks of immune cells, including *CD8B* in CD8 + T cells, *CD3E* in T cells (general), *CD9* in B cells, and *KIR2DL4* in natural killer cells (Figures 5, 6).

### Clinically Relevant Drug-Gene Interactions

Because COVID-19 patients are usually clinically treated with a variety of different drugs, the influence of drugs on these RP indicator genes should be ruled out. The DGIdb website was used to explore drug-gene interactions. Among the 59 highly accurate indicator genes, 12 were targeted by several corresponding interacting drugs, including *KCNQ1*, *TFPI*, *KMT2C*, *PGP*, *ARID5B*, *LAMB1*, *TBL1XR1*, *CDKN2D*, *KMT2E*, *FCGRT*, *PIKFYVE*, and *DHX36* (Table 4).

**TABLE 4:** Twelve indicator genes and the corresponding interacted drugs.

Genes	Interacted drugs
KCNQ1	Repaglinide, Indapamide, Dalfampridine, Insulin, Tacrolimus, Guanidine hydrochloride, Indomethacin, Dolasetron, Ezogabine, Bepridil
TFPI	Azithromycin, Lovastatin, Fulvestrant, Fenofibrate, Atorvastatin, Defibrotide
KMT2C	Anastrozole, Exemestane, Letrozole
PGP	Paclitaxel, Etoposide, Docetaxel
ARID5B	Methotrexate, Haloperidol
LAMB1	Ocriplasmin
TBL1XR1	Allopurinol
CDKN2D	Romidepsin
KMT2E	Duloxetine
FCGRT	Rozanolixizumab
PIKFYVE	Apilimod
DHX36	Manoalide

## Discussion

**FIGURE 7:**
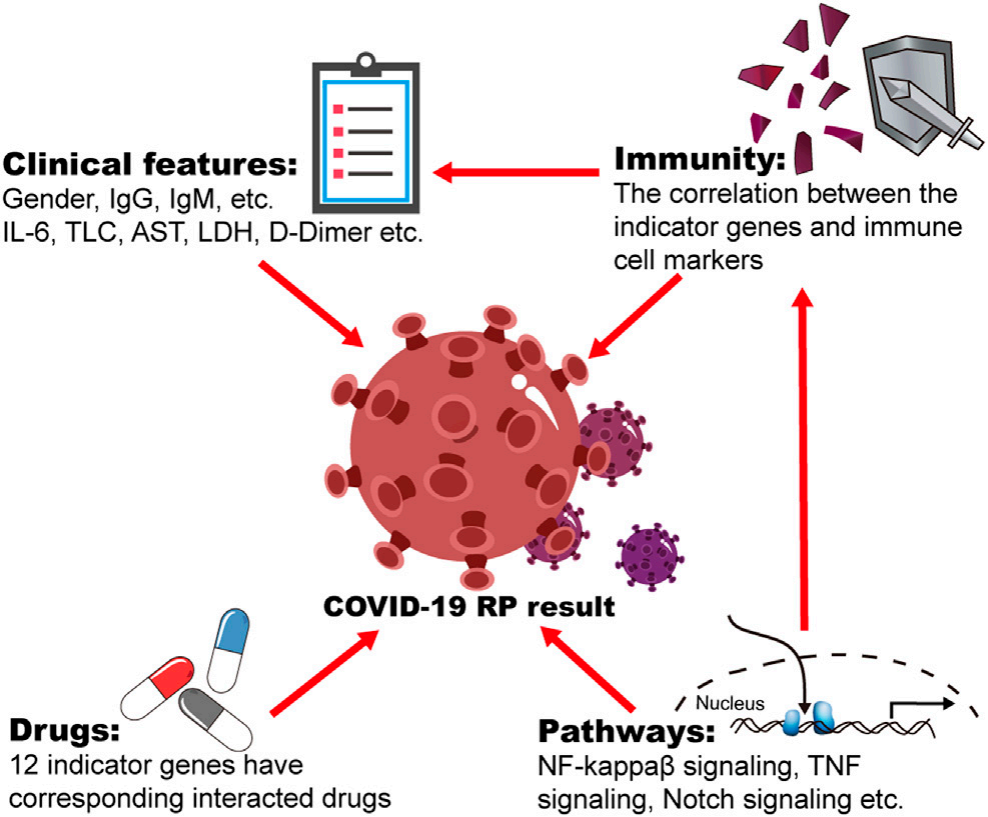
The profile of the influenced factors for COVID-19 patients with RP results.

In this study, we updated our understanding of COVID-19 RP regarding three parameters: clinical characteristics, signaling pathways, and gene signatures (Figure 7). We collected samples from COVID-19 convalescent patients and analyzed their corresponding clinical features. There were significant differences in sex and IgG and IgM titers between RP and NRP patients. To identify more accurate molecular indicators, we selected two public microarray datasets from China, GSE166253 and GSE179627, and 1,075 overlapping DEGs were screened between the NRP and RP groups. Using GO and KEGG enrichment analyses, we found that the biological functions of the overlapping DEGs were closely related to viral infection, immunity, and inflammation. The mRNA expression levels of 59 indicator genes with diagnostic values were highly correlated with the RP results, and 12 indicator genes, including *ARID5B*, *CDKN2D*, *KCNQ1*, *KMT2C*, *KMT2E*, *LAMB1*, *PGP*, *TBL1XR1*, *TFPI*, *FCGRT*, *PIKFYVE*, and *DHX36* have corresponding drug interactions. Furthermore, these indicator genes were substantially associated with gene markers of immune cells.

Among all clinical patients from whom samples were collected, 18.6% showed RP results in convalescent patients, similar to results in many previous studies (Zhu et al., 2020; Liu et al., 2021), suggesting that the RP result is a universal phenomenon. Using risk analysis, we found that the RP rate of women was higher than that of men, which is consistent with other studies (Liu et al., 2021; Wu et al., 2021). One possible reason might be that sex hormones act as important modulators of immune responses, causing different reactions to viral reinfection (Roved et al., 2017). Sex steroids, namely estrogens, progesterone, and androgens, are effective immune-modulators. The different concentrations of sex steroids between male and female are likely to influence COVID-19 immune activation and inflammatory responses (Mauvais-Jarvis et al., 2020). Moreover, previous studies illuminated that in COVID-19, testosterone contributed to inhibition of pro-inflammatory cytokines, augmentation of anti-inflammatory cytokines, thereby modulating the immune response (Pozzilli and Lenzi, 2020; Al-Kuraishy et al., 2021). In addition, our results indicated that the titers of IgG and IgM were higher in the RP groups, which may be triggered by the remaining viral RNA (Liu et al., 2021). Several other clinical risk factors associated with COVID-19 RP have been previously documented, such as age, LYM count, IL-6, AST, and D-dimer, which were not confirmed in our study. Table 2 compare the risk factors of the clinical characteristics between RP and NRP patients. Surprisingly, in previous studies, results regarding these risk factors were conflicting. For example, Liu et al. (2021). indicated that men had a lower risk than women of having a re-positive test result (*p* < 0.05), and it was confirmed in our study. However, Liu et al. (2021). analyzed that gender was no significantly different (*p* > 0.05) (Luo et al., 2021). The possible reasons for these contradictory results are that collection of clinical information was various. Also, several studies were based on a rather small cohort from a single medical center. Together, these results suggest that COVID-19 RP is likely to be a complex clinical event, and more accurate and reliable molecular indicators are urgently required for diagnosis.

To determine the molecular characteristics predicting RP patients, we compared two gene expression profile databases of peripheral blood cells from patients with RP and NRP. Using GO and KEGG pathway enrichment analyses, we found that 1,075 overlapping DEGs were related mainly to antigen processing, immune modulation, and inflammatory reactions, such as Notch, NF-кB, and mTOR signaling pathways. The NF-кB signaling pathway stimulates pro-inflammatory cytokines and chemokines, which play an important role in regulating the immune response (Kircheis et al., 2020; Montalvo Villalba et al., 2020). The Notch signaling pathway acts as a key regulator of innate immune and inflammatory responses, and it is associated with inflammatory conditions (Shang et al., 2016). The effector responses of innate immune cells can be controlled and shaped by the mTOR signaling pathway, which regulates cytokine responses and antigen presentation (Weichhart et al., 2015). Other pathways are also involved in the regulation of inflammatory cytokine responses, antigen presentation, and cellular metabolism. As these DEGs were mostly related to biological processes associated with the clinical consensus of COVID-19, we believe that they have potential and promising clinical value for the prediction of RP patients.

Because the risk factors reported in recent literature are inconsistent with each other, we conducted a more specific analysis at the gene level to discover more accurate and reliable signatures for RP patients. We found that 59 indicator genes had high correlation coefficients in patients with RP. In addition, most of these genes influenced the risk of an RP result and overlapped in the two RP-related GEO datasets; among which, 12 genes were targeted by several drugs and significantly correlated with the hallmarks of immune cells. One remarkable gene PIKFYVE was identified in PBMCs, but it could also play a essential role in preventing excessive lung inflammation through regulating alveolar macrophage function (Kawasaki et al., 2017). In addition, apilimod, the PIKFYVE inhibitor, has been found to antagonize replication of SARS-CoV-2 in iPSC-derived pneumocyte-like cells (Riva et al., 2020). Therefore, PIKFYVE might exhibit antiviral in the infected lung cells. It is possible that false negatives may be detected in COVID-19 patients who have other underlying diseases after using medicines corresponding to these genes. Although certain genes have not been previously reported to be associated with COVID-19 RP, they have been implicated in viral infections, inflammation, and immunity. For example, tissue factor pathway inhibitor (*TFPI*), which modulates a key anticoagulant pathway, can be impaired during inflammation (Levi and van der Poll, 2005). *KMT2C*, widely known as *MLL3*, activates the NF-κB pathway, inhibits inflammatory diseases, and regulates the secretion of inflammatory cytokines (Fan et al., 2021). *KMT2E* is a gene related to lysine degradation in mono-CD14^+^ cells and has been shown to be downregulated in severe COVID-19 patients (Qi et al., 2021).

Several factors influence COVID-19 RP, making it difficult to clarify the underlying mechanism. In this study, we identified relevant characteristics of RP based on clinical features, signaling pathways, and molecular biomarkers. However, due to the limited number of patient samples, some confounding factors, such as virus residue, sensitivity of test reagents, and sampling methods, were not included in the study. Although there were 1,075 overlapping DEGs between RP and NRP samples in the two GEO datasets, their clustering patterns were not the same. Generally, each cluster classification represents a pathway, or even a clinical feature, and it may not be feasible to locate consistent diagnostic features in different databases from the perspective of clinical features and signal pathways. Furthermore, there are many candidate molecular diagnostic genes that have the advantage of early detection and sensitivity, but the impact of other factors, such as drug treatment and underlying diseases, on these diagnostic indicators should be excluded.

## Conclusion

In the present study, we showed that sex and IgG and IgM titers in blood were the most distinguishing factors between RP and NRP patients. In addition, DEGs were mainly enriched in antigen recognition, inflammatory modulation, and immune responses. Co-expression networks from two mutually verified RP datasets were constructed; among which, 59 overlapping indicator genes were highly correlated with RP results. Therefore, in addition to long-term isolation and multiple nucleic acid tests, transcriptional sequencing to obtain the signatures of these indicator genes is a complementary strategy for the early and reliable diagnosis of RP patients. However, a limitation of the present study is that we did not measure infection titers in RP patients, which prevents us from evaluating the production of infectious virions. Consequently, it was impossible to confirm that whether RP patients had actively replicating virus infection.

## Data Availability

The original contributions presented in the study are included in the article/Supplementary Material, further inquiries can be directed to the corresponding authors.
