# Supplementary material for: Clinical and Gene Features of SARS-CoV-2-Positive Recurrence in Patients Recovered From COVID-19
Source: Front Mol Biosci. 2022 Jun 8;9:875418. doi: 10.3389/fmolb.2022.875418 (PMC9217101; doi:10.3389/fmolb.2022.875418)
Supplement: Supplementary file 1 [file DataSheet1.pdf]

Supplementary Material

1 Supplementary Figures

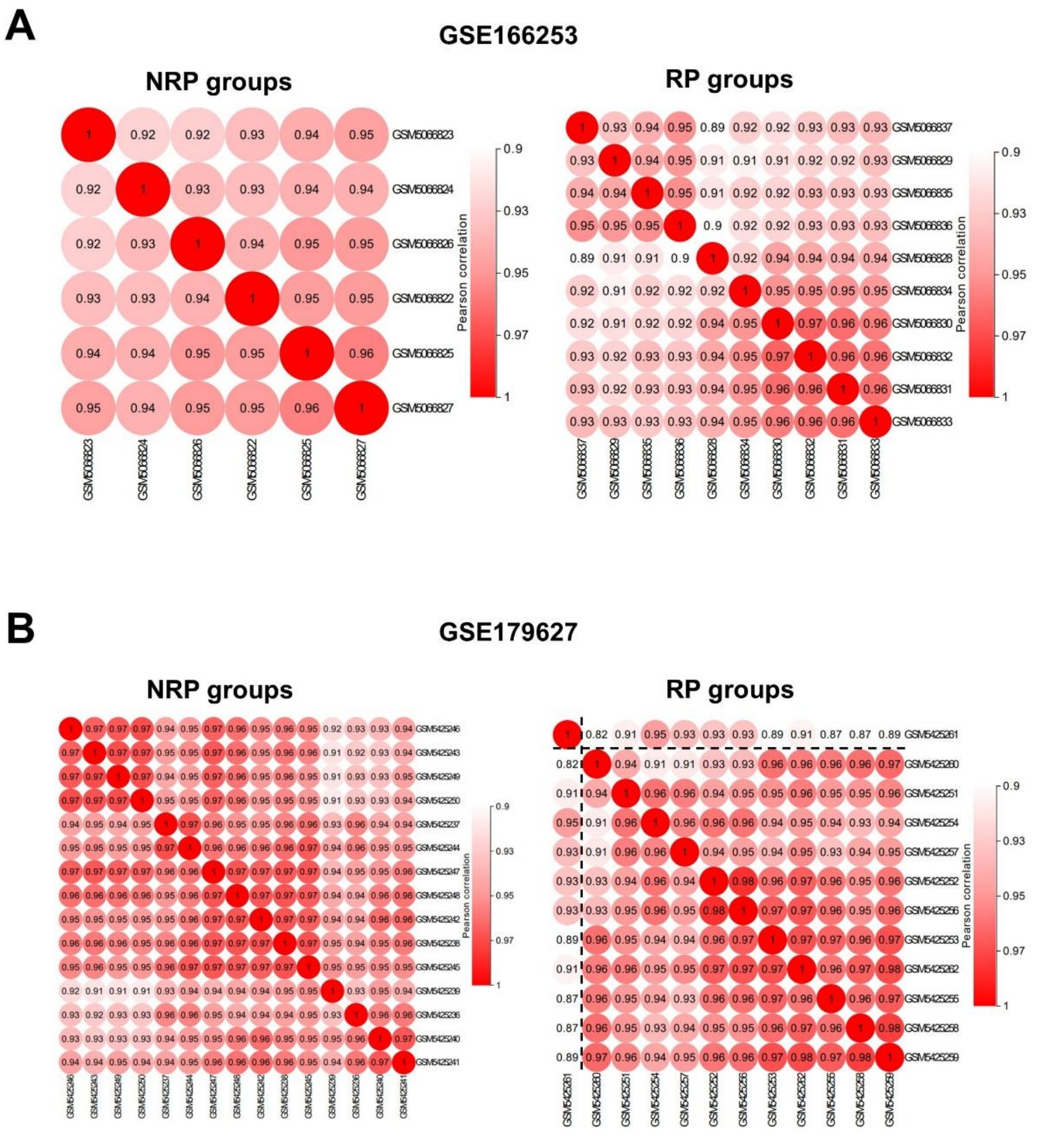

Supplementary Figure 1. The determination of analyzable samples is based on the correlation of genes.

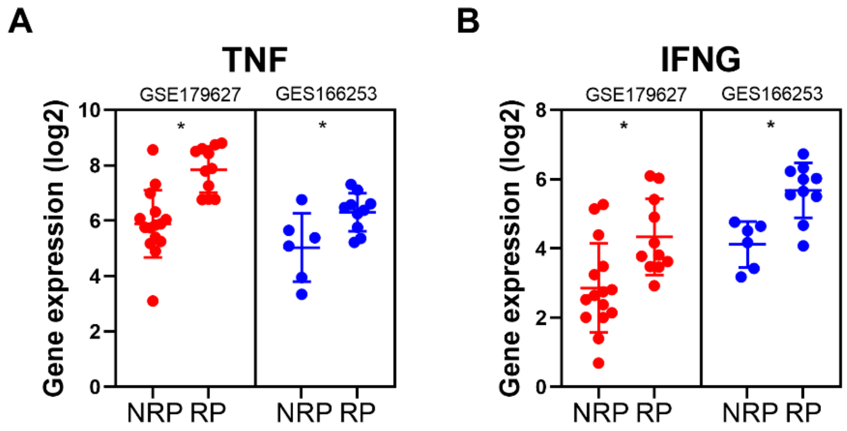

Supplementary Figure 2. The expression of TNF and IFNG in two datasets.
